# Hyperoxia provokes a time- and dose-dependent inflammatory response in mechanically ventilated mice, irrespective of tidal volumes

**DOI:** 10.1186/s40635-017-0142-5

**Published:** 2017-05-26

**Authors:** Hendrik J. F. Helmerhorst, Laura R. A. Schouten, Gerry T. M. Wagenaar, Nicole P. Juffermans, Joris J. T. H. Roelofs, Marcus J. Schultz, Evert de Jonge, David J. van Westerloo

**Affiliations:** 1 Department of Intensive Care Medicine, Leiden University Medical Center, Post Box 9600, 2300 RC Leiden, The Netherlands; 2 Department of Anesthesiology, Leiden University Medical Center, Leiden, The Netherlands; 3 Laboratory of Experimental Intensive Care and Anesthesiology, Academic Medical Center, Amsterdam, The Netherlands; 4 Department of Intensive Care Medicine, Academic Medical Center, Amsterdam, The Netherlands; 5 Department of Pediatrics, Laboratory of Neonatology, University Medical Center Leiden, Leiden, The Netherlands; 6 Department of Pathology, Academic Medical Center, Amsterdam, The Netherlands

**Keywords:** Hyperoxia, Mechanical ventilation, mice, VILI, Inflammation, Oxygen toxicity

## Abstract

**Background:**

Mechanical ventilation and hyperoxia have the potential to independently promote lung injury and inflammation. Our purpose was to study both time- and dose-dependent effects of supplemental oxygen in an experimental model of mechanically ventilated mice.

**Methods:**

Healthy male C57Bl/6J mice, aged 9–10 weeks, were intraperitoneally anesthetized and randomly assigned to the mechanically ventilated group or the control group. In total, 100 mice were tracheotomized and mechanically ventilated for either 8 or 12 h after allocation to different settings for the applied fractions of inspired oxygen (FiO_2_, 30, 50, or 90%) and tidal volumes (7.5 or 15 ml/kg). After euthanisation arterial blood, bronchoalveolar lavage fluid (BALf) and tissues were collected for analyses.

**Results:**

Mechanical ventilation significantly increased the lung injury score (*P* < 0.05), mean protein content (*P* < 0.001), and the mean number of cells (*P* < 0.01), including neutrophils in BALf (*P* < 0.001). In mice ventilated for 12 h, a significant increase in TNF-α, IFN-γ, IL-1β, IL-10, and MCP-1 (*P* < 0.01) was observed with 90% FiO_2_, whereas IL-6 showed a decreasing trend (*P* for trend = 0.03) across FiO_2_ groups. KC, MIP-2, and sRAGE were similar between FiO_2_ groups. HMGB-1 was significantly higher in BALf of mechanically ventilated mice compared to controls and showed a gradual increase in expression with increasing FiO_2_. Cytokine and chemokine levels in BALf did not markedly differ between FiO_2_ groups after 8 h of ventilation. Differences between the tidal volume groups were small and did not appear to significantly interact with the oxygen levels.

**Conclusions:**

We demonstrated a severe vascular leakage and a pro-inflammatory pulmonary response in mechanically ventilated mice, which was enhanced by severe hyperoxia and longer duration of mechanical ventilation. Prolonged ventilation with high oxygen concentrations induced a time-dependent immune response characterized by elevated levels of neutrophils, cytokines, and chemokines in the pulmonary compartment.

**Electronic supplementary material:**

The online version of this article (doi:10.1186/s40635-017-0142-5) contains supplementary material, which is available to authorized users.

## Background

Supplemental oxygen administration is essential to enhance survival in respiratory impaired and mechanically ventilated patients. Inspiratory fractions of oxygen (FiO_2_) typically exceed concentrations of atmospheric air and are frequently applied for prolonged periods during mechanical ventilation in patients suffering from severe respiratory distress. However, both mechanical ventilation and hyperoxia can promote lung injury and induce adverse effects through diverse mechanisms. Clinical studies have retrospectively shown associations between arterial hyperoxia and poor outcomes during specific cardiovascular, neurological, respiratory, and traumatic events [1–4]. Accumulating evidence indicates a U-shaped survival curve of critically ill and mechanically ventilated patients in relation to arterial oxygen levels in the first 24 h of admission [5–8].

Impaired lung function may be caused by the adverse hemodynamic effects that are mainly imputed to direct vasoconstrictive actions of high oxygen concentrations, and atelectasis which may be aggravated by local and systemic inflammatory responses. These responses have repeatedly been documented in rodents following hyperoxic exposure in inhalation chambers [9–15]. Although hyperoxia has been suggested to induce time-dependent inflammatory responses [10], studies in animals are usually restricted to periods of up to 6 h of mechanical ventilation, limiting its clinical applicability [16–19]. Furthermore, the interaction between mechanical ventilation and concurrent hyperoxia may transcend lung injury by alveolar distention alone [9, 17, 20–22]. Given that oxygen therapy cannot altogether be avoided, we aimed to increase knowledge on the host response to different levels of oxygen. Hypothesizing that hyperoxia induces a dose-dependent gradual inflammatory response that may be aggravated by prolonged periods of mechanical ventilation, our purpose was to induce hyperoxia in mice and study both time- and dose-dependent inflammation effects of supplemental oxygen during prolonged ventilatory support with protective and injurious tidal volumes.

## Methods

The Animal Care and Use Committee of the Academic Medical Center of the University of Amsterdam, The Netherlands, approved the study protocol in accordance with applicable research and ethical protocols. Animal procedures were performed in consistence with Institutional Standards for Care and Use of Laboratory Animals.

### Animals

Healthy male C57Bl/6J mice were obtained from Charles River (Maastricht, The Netherlands) and housed in a temperature- and light-controlled room. The animals were acclimatized at the animal facility for at least 7 days and had free access to rodent chow and water. Animal welfare was warranted throughout the experiment [23]. Conscious animals were injected twice for intraperitoneal prehydration and anesthesia induction. Thereafter, mice were regularly checked on pain stimuli and discomfort. The titration scheme for adequate anesthesia was determined in pilot experiments.

### Design

At baseline, 109 mice aged 9–10 weeks (20–28 g) were intraperitoneally prehydrated with a bolus of 1 ml normal saline and randomly assigned to the mechanical ventilation (MV) group (*n* = 100) or the control group (*n* = 9). All mice in the MV group were randomized to subgroups (*n* = 8–9 per subgroup) by allocating different settings for the applied fractions of inspired oxygen (FiO_2_ = 30, 50, or 90%), tidal volume (TV = 7.5 or 15 ml/kg), and MV duration (8 or 12 h). The control group mice were spread over multiple days along with the instrumentation of the MV groups during the whole experimental period.

Experimental procedures have been described in detail previously [24, 25]. One hour after prehydration, mice assigned to the MV groups (*n* = 8–9 per group) were anesthetized with a 0.15–0.21 ml intraperitoneal bolus of 126 mg/kg ketamine (Eurovet Animal Health BV, Bladel, The Netherlands), 0.1 mg/kg dexmedetomidine 0.5 mg/ml (Elanca Animal Health, Houten, The Netherlands), 0.5 mg/kg atropine sulfate (Centrafarm BV, Etten-Leur, The Netherlands), and 5 ml/kg 0.9% saline. Maintenance anesthesia was injected hourly through an intraperitoneal catheter (PE 10 tubing, BD, Breda, The Netherlands) and consisted of 36 mg/kg ketamine, 0.02 mg/kg dexmedetomidine, 0.075 mg/kg atropine sulfate, and 9.45 ml/kg 0.65% saline. A 1:5 mix of 0.65% saline and 8.4% sodium bicarbonate was intraperitoneally administered through the catheter every 30 min in order to compensate for fluid loss and maintain physiological bicarbonate levels [25]. Body temperature was strictly controlled between 36.5 and 37.5 °C. Systolic blood pressure and heart rate were noninvasively measured using a murine tail pressure cuff with pulse transducer and monitored on a data acquisition system (LabChart, ADInstruments Ltd, Oxford, UK). Tidal volumes were monitored using a calibrated pneumotachometer (tracheal cannula OD = 1.3 mm, PTM type 378/0.9, HSE-Harvard Apparatus GmbH, March-Hugstetten, Germany) and respiration data acquisition software (BDAS, HSE-Harvard Apparatus GmbH).

Anesthetized mice were tracheotomized, and a Y-tube connector (OD 1.0 mm, ID 0.6 mm) was surgically inserted in the trachea and fixed above the carina. Subsequently, animals were placed on a heating plate in supine position and connected to the ventilator (Babylog 8000 plus, Dräger Medical, Lübeck, Germany). Ventilator settings were pre-determined in pilot experiments and targeted at normal acid–base balance [24]. Ventilators were pressure-controlled and set to deliver low tidal volume (LTV, 7.5 ml/kg) or high tidal volume (HTV, 15 ml/kg). In both ventilation strategies, positive end-expiratory pressure (PEEP) levels were set at 3 cmH_2_O and the inspiration to expiration ratio at 1:2.8. Respiratory rates were controlled at 160 (LTV) or 52 (HTV) breaths per minute. Recruitment maneuvers were performed every 30 (LTV) or 60 (HTV) minutes by means of inspiratory holds with a pressure of 20 mbar during 5 s.

Immediately after randomization, ventilators were adjusted to the assigned settings by an independent biotechnician. Inspiratory pressures were adjusted and regulated to achieve appropriate TV throughout the experiment. At the end of the experiment, ventilated mice were euthanized by withdrawing blood from the carotid artery. Researchers were blinded for administered FiO_2_ levels during the experimental procedures. The allocation code of randomization was supplied by the time all data and assay results were collected.

### Measurements

After euthanization, arterial blood was collected in heparin-coated syringes and used for blood gas analysis (Rapidpoint 405, Siemens Healthcare, Tarrytown, NY, USA). Lungs were resected en bloc, and the right lung was instilled with normal saline (3 × 0.5 ml) to obtain bronchoalveolar lavage fluid (BALf), which was used for automated cell counting (Z2 Beckman Coulter Counter, Brea, USA). Differential counts were performed on Giemsa stained cytospin slides. BALf was centrifuged, and the supernatant was stored at −80 °C for assessment of protein levels and cytokines. The left lung was weighed and thereafter fixed in 4% formalin and embedded in paraffin. Lung sections were stained with hematoxylin eosin (H&E) to analyze lung histopathology. A dedicated pathologist determined the histopathological lung injury score on a nominal scale by the sum of the score for four pathologic parameters: edema, hemorrhage, interstititial cell infiltration, and hyaline membranes as described previously [24]. Relative lung weight, expressed as lung weight corrected for total body weight at baseline, was used as surrogate for lung tissue edema.

Cytokines (IL-1β, IL-6, IL-10, MCP-1, MIP-2, KC, TNF-α, IFN-γ) were measured in BALf by Luminex (Merck Millipore Chemicals BV, Amsterdam, The Netherlands). High-mobility group box-1 (HMGB-1, IBL International BV, Amersfoort, The Netherlands) and the soluble receptor for advanced glycation end products (sRAGE, R&D Systems, Abingdon, UK) were determined by enzyme-linked immunosorbent assays (ELISA) according to the manufacturer’s protocols. Total protein levels were determined in serum and BALf (Oz Biosciences, Marseille, France), using bovine serum albumin as reference.

The right lung was used for total RNA isolation from tissue homogenates (RNA-Bee, Tel-Test Inc, Bio-Connect BV, Huissen, The Netherlands), first-strand cDNA synthesis (SuperScript Choice System, Life Technologies, Breda, The Netherlands), and real-time quantitative PCR (TNF-α, IL-6, MMP-12, MCP-1, TF, PAI-1), using β-actin as a housekeeping gene reference, were performed on a LightCycler 480 (Roche, Almere, The Netherlands) of the Leiden Genome Technology Center (Leiden, The Netherlands) as described previously [26].

### Statistical analysis

The partial pressure of arterial oxygen (PaO_2_) at the end of the experiment was defined as the primary outcome. Inflammatory markers and markers of lung injury were assessed as secondary outcomes. Based on previous pilot results and with a group size of eight animals per group the Wilcoxon rank-sum test ensures 80% power using a two-sided significance level of 0.05 to detect an estimated effect size of 1.85 that the observed parameter differs between groups. With an anticipated dropout rate of one per group, nine animals were initially assigned to each group. Differences between study groups were tested with one-way analysis of variance or Kruskal Wallis as appropriate. Cuzick’s test was used to test for trends across different FiO_2_ groups. Statistical analyses were performed using R version 3.2.1 (R Foundation for Statistical Computing, Vienna, Austria) and STATA/SE 10.1 (StataCorp LP, College Station, TX, USA).

## Results

All mice survived mechanical ventilation with the applied settings in a volume-targeted approach (Table 1). Mean systolic blood pressures decreased gradually during 8 or 12 h of mechanical ventilation (145 to 90 mmHg) and were slightly higher in the 90% FiO_2_ group (Table 1, Additional file 1 Figure S1). Heart rates and body temperatures remained stable throughout the experiment.

**Table 1:** Ventilation and hemodynamic parameters

	8 h			12 h		
	30%	50%	90%	30%	50%	90%
LTV						
TV (μl)	181 (0)	182 (0)	179 (0)	176 (0)	178 (1)	176 (0)
P_insp_ (mbar)	13 (0)	14 (0)	12 (0)	12 (0)	13 (0)	12 (0)
SBP (mmHg)	116 (7)	128 (7)	123 (5)	103 (4)	102 (5)	112 (4)
HTV						
TV (μl)	351 (0)	379 (1)	372 (1)	366 (2)	371 (1)	360 (1)
P_insp_ (mbar)	20 (0)	21 (0)	20 (0)	20 (0)	20 (0)	20 (0)
SBP (mmHg)	114 (6)	136 (8)	124 (7)	104 (4)	110 (6)	113 (5)

Data are means ± SD. TV = tidal volume, P_insp_ = inspiratory pressure, SBP = systolic blood pressure. All indicated parameters were measured hourly.

### Oxygenation and ventilation

**Fig. 1:**
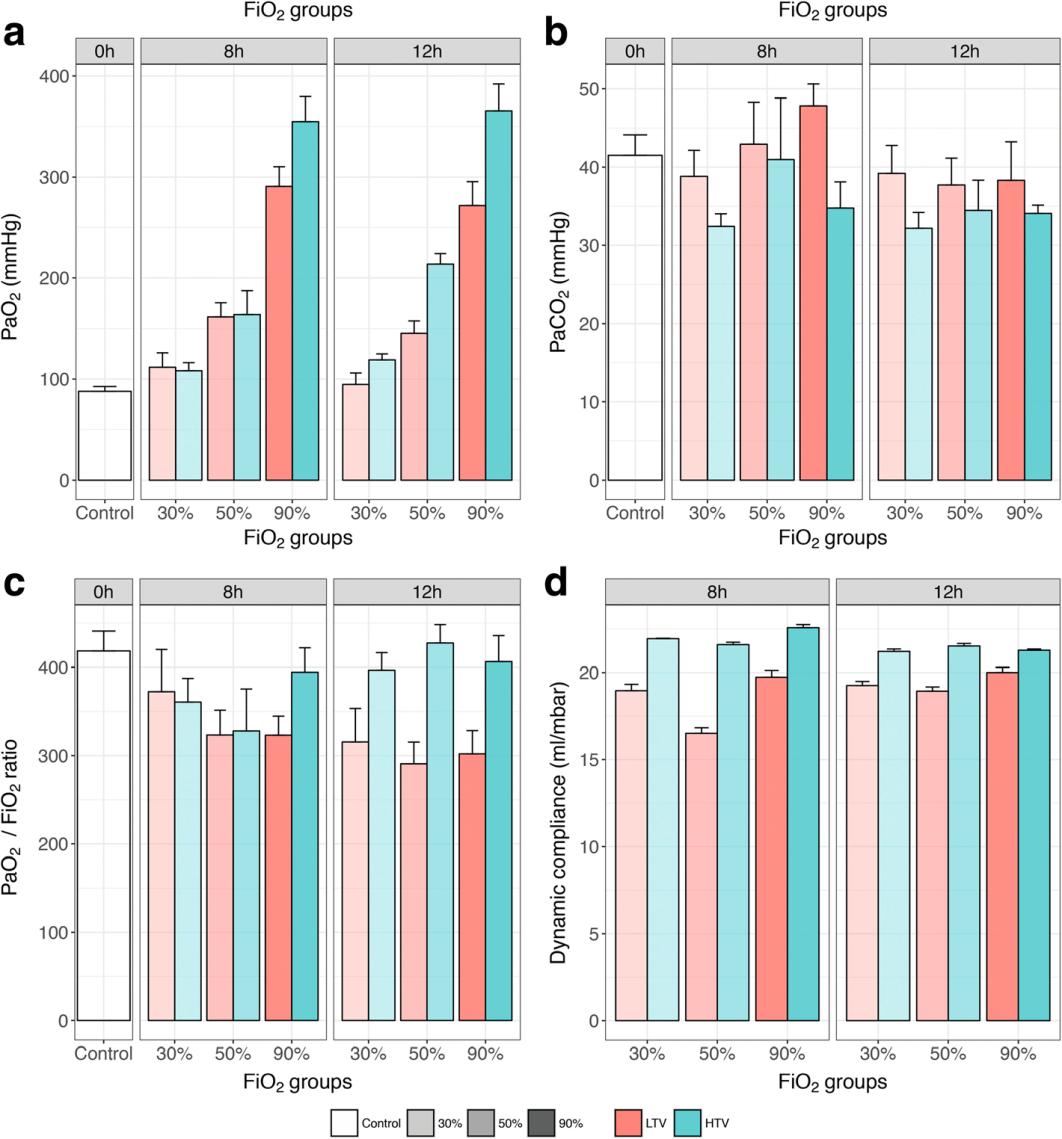
Arterial oxygenation and ventilation parameters. Data are means ± SEM. Arterial oxygenation in carotid blood (**a**, *upper left* panel), arterial carbon dioxide in carotid blood (**b**, *upper right* panel), PaO_2_/FiO_2_ ratio (**c**, lower left panel), and dynamic compliance (**d**, *lower right* panel). *Facets* within the panels represent mechanical ventilation time. *Different colors* represent different tidal volume groups, and *different transparency levels* represent different FiO_2_ groups. *0h* no mechanical ventilation time, control group; *8h* 8 h of mechanical ventilation, *12h* 12 h of mechanical ventilation, *LTV* *low* tidal volumes, *HTV* high tidal volumes. Dynamic lung compliance (tidal volume size/(peak inspiratory pressure − PEEP) was measured hourly. PaO_2_ and PaCO_2_ were measured once in the arterial blood gas sample taken from the carotid artery at the end of the experiment

The partial pressure of oxygen in the carotid arterial blood at the end of mechanical ventilation was distinctly higher with increasing fractions of supplied oxygen (*P* < 0.001, Fig. 1a). Partial pressure of arterial carbon dioxide (PaCO_2_) was in general lower for the HTV groups but did not show a trend across FiO_2_ groups (Fig. 1b). The PaO_2_/FiO_2_ ratio decreased for mechanically ventilated mice in comparison to controls and was markedly higher for HTV groups after 12 h of mechanical ventilation (Fig. 1c). Dynamic lung compliance decreased gradually over the study interval for all study groups, but the decrease was nearly complete after 3 h of mechanical ventilation and was larger for the HTV groups (Fig. 1d).

### Markers of lung injury

**Fig. 2:**
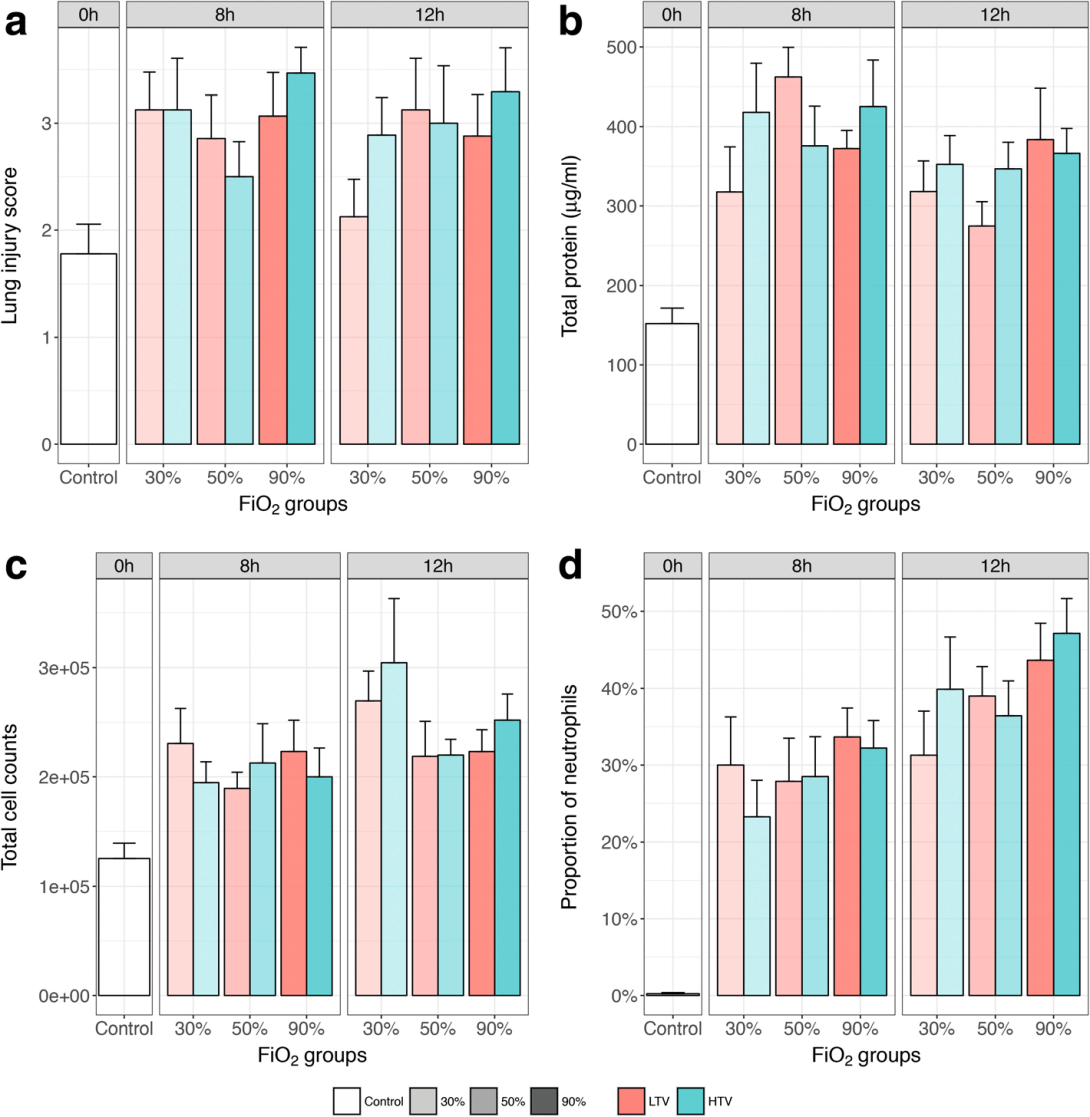
Markers of lung injury in BALf after indicated study interval. Data are means ± SEM. Lung injury score (**a**, *upper left* panel), total protein content (**b**, *uppe*r right panel), total cell counts (**c**, *lower left* panel), and proportion of neutrophils (**d**, *lower right* panel) in BALf obtained after the study interval. *Facets* within the panels represent mechanical ventilation time. *Different colors* represent different tidal volume groups, and *different transparency levels* represent different FiO_2_ groups. *0h* no mechanical ventilation time, control group; *8h* 8 h of mechanical ventilation, *12h* 12 h of mechanical ventilation, *LTV* *low* tidal volumes, *HTV* high tidal volumes

Mechanical ventilation significantly increased the lung injury score (Fig. 2a, 1.6-fold at 8 h, *P* < 0.01; and 1.5-fold at 12 h, *P* < 0.05), mean protein content (Fig. 2b, 2.6-fold at 8 h, *P* < 0.001; and 2.2-fold at 12 h, *P* < 0.001), and the mean number of cells (Fig. 2c, 1.7-fold at 8 h, *P* < 0.01; and 2.0-fold at 12 h, *P* < 0.001), including neutrophils in BALf (Fig. 2d, 132-fold at 8 h, *P* < 0.001; and 180-fold at 12 h, *P* < 0.001), demonstrating vascular leakage and inflammation as a result of mechanical ventilation even at relatively low hyperoxic conditions of 30% FiO_2_ and low tidal volumes. Increased hyperoxia up to 90% FiO_2_ did not further increase protein content or the total number of cells in BALf but showed an increased trend in the percentage of neutrophils towards higher FiO_2_ levels (*P* for trend = 0.03). Histopathology showed a decrease in air restraint, suggesting progressive alveolar collapse, with higher oxygen levels (Additional file 1 Figure S2), but this was not translated in a significant difference in the lung injury score between the different FiO_2_ groups (Fig. 2a).

### Markers of inflammation

**Fig. 3:**
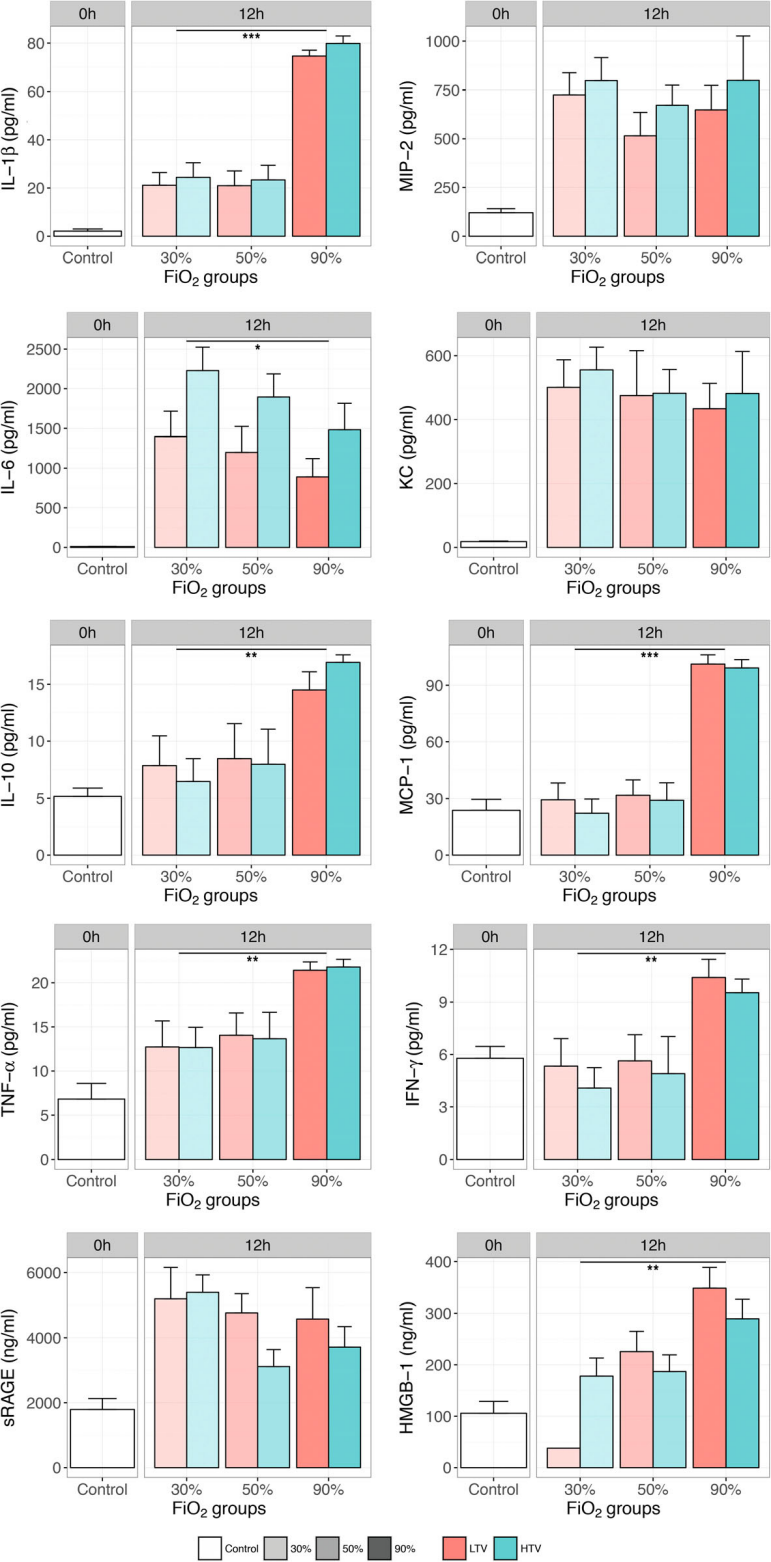
Inflammatory mediators in BALf in controls and after 12 h of mechanical ventilation. Cytokine and chemokine levels in BALf obtained after 12 h of mechanical ventilation. Data are means ± SEM. *Facets* within the panels represent mechanical ventilation time. *Different colors* represent different tidal volume groups, and *different transparency levels* represent different FiO_2_ groups. *0h* no mechanical ventilation time, control group; *12h* 12 h of mechanical ventilation, *LTV* low tidal volumes, *HTV* high tidal volumes. Cuzick’s *P* for trend in increasing oxygen levels at 12 h of mechanical ventilation: IL-1β, <0.001; MIP-2, 0.27; IL-6, 0.03; KC, 0.22; IL-10, 0.001; MCP-1, <0.001; TNF-α, 0.001; IFN-γ, 0.001; sRAGE, 0.11; HMGB-1, 0.001. *P for trend <0.05; **P for trend <0.01; ***P for trend <0.001

Cytokine and chemokine levels in BALf increased at 8 and 12 h after mechanical ventilation but did not markedly differ between FiO_2_ groups at 8 h of ventilation (*P* for trend >0.05, Additional file 1 Figure S3). In mice ventilated for 12 h, a significantly increasing trend in TNF-α, IFN-γ, IL-1β, IL-10, and MCP-1 (Fig. 3, *P* for trend <0.01) was observed with increasing FiO_2_, whereas IL-6 showed a decreasing trend (*P* for trend = 0.03). KC, MIP-2, and sRAGE were similar between FiO_2_ groups. HMGB-1 was significantly higher in BALf of mechanically ventilated mice compared to controls and showed a gradual increase in expression with increasing FiO_2_. Almost no differences in cytokine and chemokine levels in the BALf were observed between the 30 and 50% oxygen groups.

Differences between the tidal volume groups were small (Additional file 1 Figure S4) and did not appear to significantly interact with the oxygen levels (*P* > 0.50 for the interaction term for each inflammatory mediator, except for IL-6, *P* = 0.03). Inflammatory markers were also measured at 12 h of mechanical ventilation in the serum and are shown in Additional file 1 Figure S5.

The RNA expression of selected markers showed an increased relative expression of TNF-α, IL-6, and MCP-1 in lung homogenate of mice that were mechanically ventilated with high tidal volumes compared to controls (Additional file 1 Figure 6).

## Discussion

In this experimental study, we demonstrated a severe vascular leakage and a pro-inflammatory pulmonary response in mechanically ventilated mice, which was enhanced by severe hyperoxia and longer duration of mechanical ventilation. Prolonged ventilation with high oxygen concentrations induced a time-dependent immune response characterized by elevated levels of neutrophils, cytokines, and chemokines in the pulmonary compartment.

Although most studies in mechanically ventilated animals are restricted to short exposure periods, recent experiments in mechanically ventilated rodents, rabbits, and pigs, mimicking the clinical environment of critically ill patients, showed that hyperoxia serves as an important cofactor in acute lung injury, bacterial dissemination, progression of multiple system organ dysfunction, and lethality [13–15] but can also improve organ function and attenuate tissue apoptosis during shock [27]. In our study, divergent effects were observed in the expression of selected inflammatory markers across the experimental groups, which may be explained by the complex kinetics and dynamics of the immune response due to the concurrent exposure to anesthesia, mechanical ventilation, and hyperoxia. Interaction between pro- and anti-inflammatory cytokines may contribute to the differences in cytokine levels in the experimental groups. Rapid upregulation of TNF-α was seen even in the acute phase reaction after induction of anesthesia in control mice. Cytokine concentrations may decrease after long-term hyperoxic exposure, and a fast upregulation of inflammatory action can be followed by a gradual impairment or suppression of the innate immune system [28], which may in turn make the lung more susceptible to injury and infection. Differences in cytokine levels between the FiO_2_ groups were relatively mild. We observed a strong inflammatory effect at very high oxygen concentrations (FiO_2_ 90%) while the increase from 30 to 50% did not make a large difference. Indeed, 50% of inspired oxygen may not be as detrimental as 90% as evidenced by the clear increase in neutrophils and most cytokines during 90% oxygen administration, which was also described in other studies [29–32]. In addition, ventilation with 30% may actually be a model of relative tissue hypoxia in mice following progressive lung injury during the experiment, although this was not reflected by the PaO_2_ levels. Comparison with control groups suggests that mechanical ventilation itself was very harmful, possibly not allowing additional damage by increasing oxidative stress. Both hypoxia and hyperoxia may induce oxidative stress, and relative changes in hyperoxia may trigger upregulation of the hypoxia inducible factor (HIF) [33].

Furthermore, damage-associated molecular pattern (DAMP) molecules play a key role in the inflammatory response to injury and have been suggested to modulate the effects of hyperoxia and oxidative stress [34, 35]. In this matter, HMGB-1 has been described as the archetypal chemokine that is upregulated by the innate immune system in response to cell stress [36]. We also observed increased levels of this protein in the lungs of mechanically ventilated mice, particularly after concurrent exposure to high FiO_2_. The overall protein content did not significantly increase with severe hyperoxia, but this interpretation may be limited as we did not correct the protein in BALf for urea (epithelial lining fluid). The expression of most cytokines in BALf was not essentially different than in the serum, which may be a result of extended duration of mechanical ventilation causing systemic inflammation. In line with previous experiments, the presence of cytokines with short term ventilation was definitely more pronounced in the pulmonary compartment compared to circulating blood of rodents [25, 37].

Our study and others generated conflicting data regarding the inflammatory response after hyperoxic ventilation describing both pro- [15, 17, 38, 39] and anti-inflammatory [40, 41] responses. Kiers et al. recently demonstrated that, in the absence of systemic inflammation, short-term hyperoxia without mechanical ventilation does not result in increased levels of inflammatory cytokines, neutrophil phagocytosis, nor ROS generation in both mice and healthy volunteers [42]. In the present study, the expression of inflammatory markers was shown to be divergent after mechanical ventilation and with increasing FiO_2_, which is consistent with previous research [16]. Bailey et al. concluded that prolonged mechanical ventilation of healthy rat lungs with a physiological strategy can contribute to the inflammatory response and cause alterations to pulmonary surfactant [43]. Lung injury due to continuous hyperoxic exposure has also been shown to be dose-dependent in rats [12, 15].

Some clinical scenarios may dictate non-protective ventilation, both with high pressures and high levels of inspired oxygen in patients with heavily injured lungs. However, it is not exactly known whether this combination works synergistically in causing lung injury. Our data do not imply such an “add-on effect,” but the discrepancy with a previous study [20] may be explained by the use of lower tidal volumes in our study. It is also possible that the extended duration of mechanical ventilation alone was enough to cause ventilator-induced lung injury (VILI) without an additional effect of tidal volume size. An alternative explanation may be that lower respiratory rates compensated for high tidal volumes, while higher respiratory rates increased the risk of lung injury in the lower tidal volume groups. Also, some of the deleterious properties of hyperoxia may be overcome by applying PEEP, as it may counteract alveolar collapse from progressive nitrogen washout and mitigate the effects of atelectrauma [44].

In our study, lung injury scores did not reveal any histopathological difference between study groups. However, in previous work using a high tidal volume strategy with zero PEEP, total histopathology scores were shown to be higher compared to low tidal volumes and 3 cmH_2_O PEEP with a marginal additive effect of ventilation duration [24]. This was in accordance with a study reporting that pre-exposure to hyperoxia increases the susceptibility to VILI before initiation of mechanical ventilation [22]. Others documented that short-term exposure to levels of oxygen up to 100% does not increase the changes in respiratory system mechanics induced by mechanical ventilation [16]. The progressive airway collapse and inflammation with increasing FiO_2_ may not have been severe enough to induce histopathological changes and affect lung function in our model. The striking heterogeneity that exists between experimental studies may be explained by differences in subjects (e.g., species, strains, genetical modification, age, sex), and exposure (e.g., pre-exposure, severity, duration, anesthesia, ventilation).

Although the experiments were performed according to the high standards for methodological quality of animal research [45], several limitations may apply to our experimental procedures. The lavage technique of the lungs may induce subtle differences in the dilution of returned fluid, although saline injection volumes were standardized. Storage of biological specimens was secured according to high-quality protocols. Our analyses were restricted to specific features of injurious ventilation and hyperoxia, yet other underlying mechanisms affecting reactive oxygen species and mitochondrial damage have not been considered. Other covariates such as PaCO_2_ may have influenced the results, even though this cannot be seen separate from ventilation settings and was inherent to adjusting tidal volumes and FiO_2_. Furthermore, low respiratory rates, especially in the LTV group, are subphysiological for mice and may cause relative hypercapnia.

The experimental setting may hamper translation of study results to the clinical setting. Indeed, smaller species, such as mice have different lung mechanics and immune reactions than humans [46, 47]. Healthy mice may also respond differently than critically ill patients, especially in case of injured lungs prior to the start of mechanical ventilation. Interestingly, moderate hyperoxia in mechanically ventilated patients without severe respiratory failure does not appear to increase systemic or pulmonary inflammation [48].

Further, the C57Bl/6J type mice that we used are the most widely used strain of mice for experimental research but have been shown to carry a spontaneous mutation, which can result in mitochondrial redox abnormalities and may influence the functionality of the hyperoxia defense [49].

Strengths of this study include the prolonged duration of mechanical ventilation which may be representative of the intensive care unit (ICU) setting, where hyperoxia acts as a second hit on top of VILI. Indeed, we showed that the immune response was considerably stronger at 12 h of mechanical ventilation compared to 8 h, which may imply that models applying mechanical ventilation for extended duration more accurately reflect the underlying mechanisms and long-term effects. Demonstrable lung injury may follow the inflammatory response even later than after 12 h of hyperoxic mechanical ventilation. As such, our results further accentuate that we should limit the exposure to supraphysiological oxygen levels from excessive oxygen supply when prolonged periods of mechanical ventilation are anticipated.

## Conclusions

Prolonged experimental hyperoxic mechanical ventilation was associated with a significant inflammatory response in the lung as evidenced by an influx of neutrophils in the pulmonary compartment and upregulation of specific inflammatory markers, which was not directly translated into extensive tissue lung injury or a change in lung compliance. The present experimental data may aid to determine optimal ventilator strategies in mechanically ventilated patients, but the dynamics and kinetics of hyperoxic ventilation need further exploration in order to characterize the long-term effects and investigate protective measures.

## Additional files

Additional file 1: Figure S1. Mean systolic blood pressure over the study interval. **Figure S2.** Microscopic histopathology of representative mouse lung sections after 12 h of mechanical ventilation (H&E staining, ×10 magnification). **Figure S3.** Inflammatory mediators in BALf after 8 h of mechanical ventilation. **Figure S4.** Inflammatory mediators in BALf after study interval by tidal volume size. **Figure S5.** Inflammatory mediators in serum after 12 h of mechanical ventilation. **Figure S6.** Relative RNA expression of inflammatory markers in lung homogenate after 12 h of mice that were mechanically ventilated with high tidal volumes compared to controls.

## Abbreviations

BALf: Bronchoalveolar lavage fluid
DAMP: Damage associated molecular pattern
ELISA: Enzyme-linked immunosorbent assays
FiO_2_: Fraction of inspired oxygen
HIF: Hypoxia inducible factor
HMGB-1: High-mobility group box-1
HTV: High tidal volume
ICU: Intensive care unit
IFN-γ: Interferon-γ
IL-10: Interleukin-10
IL-1β: Interleukin-1β
IL-6: Interleukin-6
KC: Kupffer Cell
LTV: Low tidal volume
MCP-1: Monocyte Chemoattractant Protein-1
MIP-2: Macrophage Inflammatory Protein-2
MV: Mechanical ventilation
PaCO_2_: Partial pressure of arterial carbon dioxide
PaO_2_: Partial pressure of arterial oxygen
PEEP: Positive end-expiratory pressure
sRAGE: soluble Receptor for Advanced Glycation End products
TNF-α: Tumor necrosis factor-α
TV: Tidal volume
VILI: Ventilator-induced lung injury
