# Supplementary material for: Hyperoxia provokes a time- and dose-dependent inflammatory response in mechanically ventilated mice, irrespective of tidal volumes
Source: Intensive Care Med Exp. 2017 May 26;5:27. doi: 10.1186/s40635-017-0142-5 (PMC5446430; doi:10.1186/s40635-017-0142-5)
Supplement: Supplementary file 1 — Mean systolic blood pressure over the study interval. Figure S2. Microscopic histopathology of representative mouse lung sections after 12 h of mechanical ventilation (H&E staining, ×10 magnification). Figure S3. Inflammatory mediators in BALf after 8 h of mechanical ventilation. Figure S4. Inflammatory mediators in BALf after study interval by tidal volume size. Figure S5. Inflammatory mediators in serum after 12 h of mechanical ventilation. Figure S6. Relative RNA expression of inflammatory markers in lung homogenate after 12 h of mice that were mechanically ventilated with high tidal volumes compared to controls. [file 40635_2017_142_MOESM1_ESM.docx]

SUPPLEMENTAL DATA

**Supplemental figure 1.** Mean systolic blood pressure over the study interval


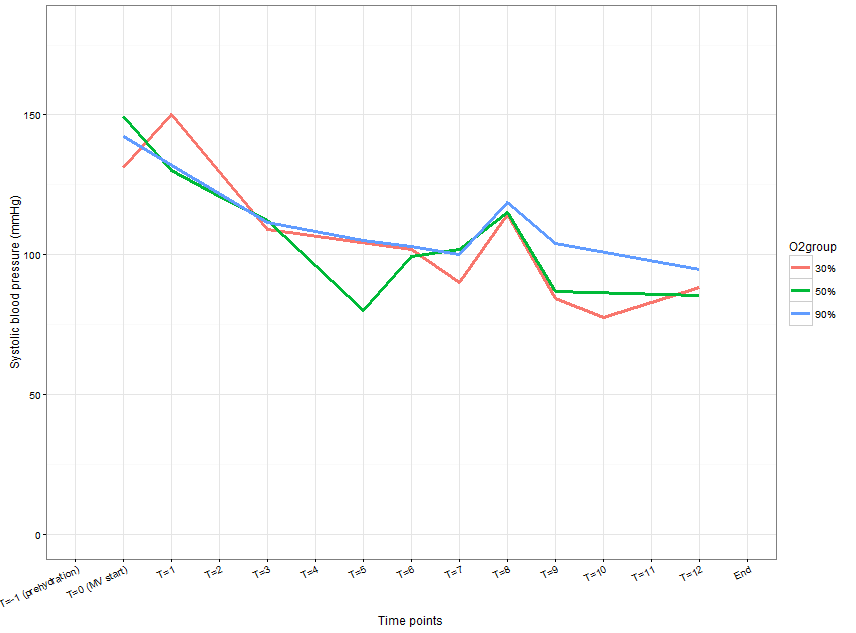


**Supplemental figure 2.** Microscopic histopathology of representative mouse lung sections after 12 hours of mechanical ventilation (H&E staining, 10x magnification)

| **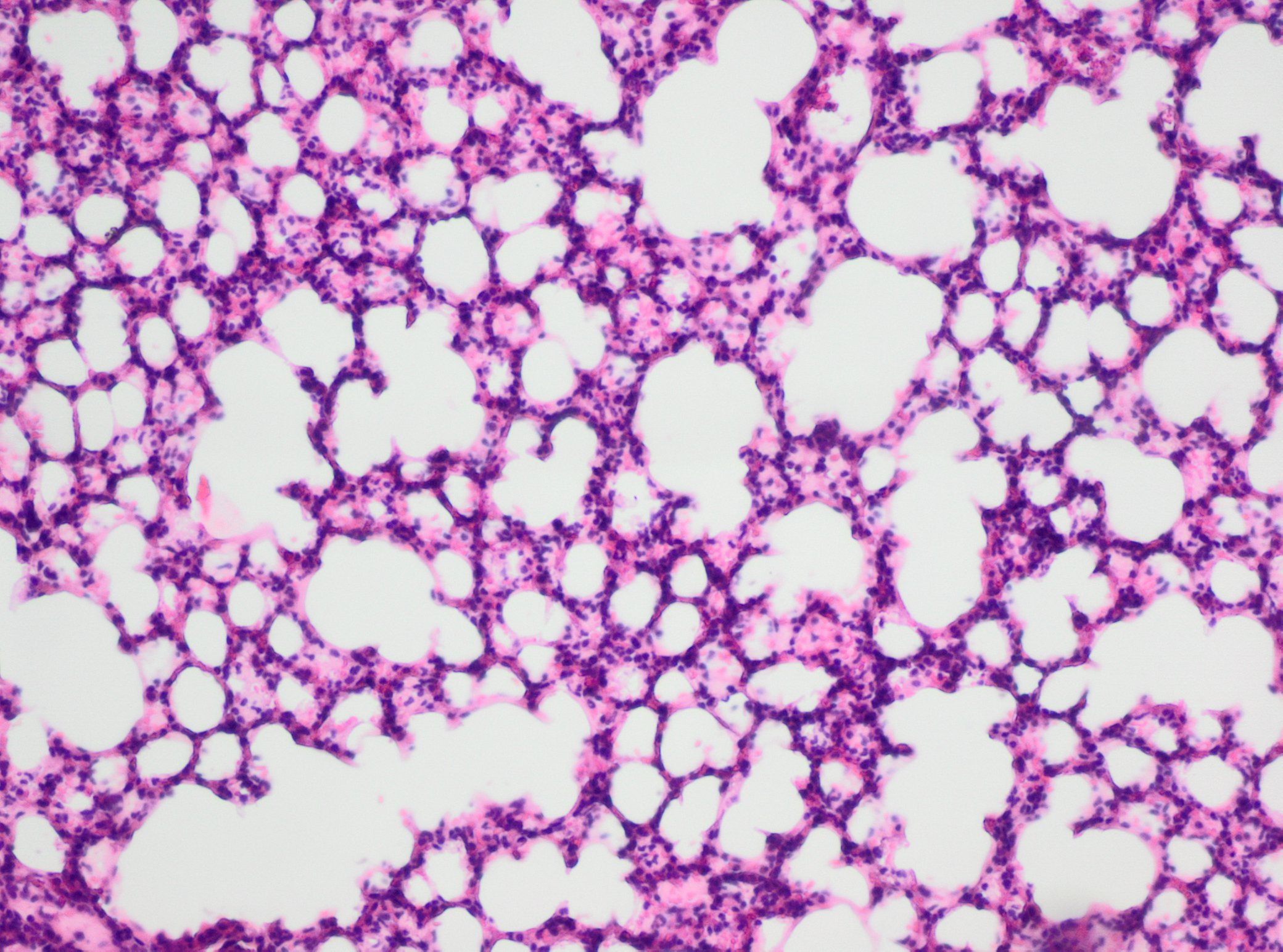**  **Fig. 2a** Control | | |
| --- | --- | --- |
| **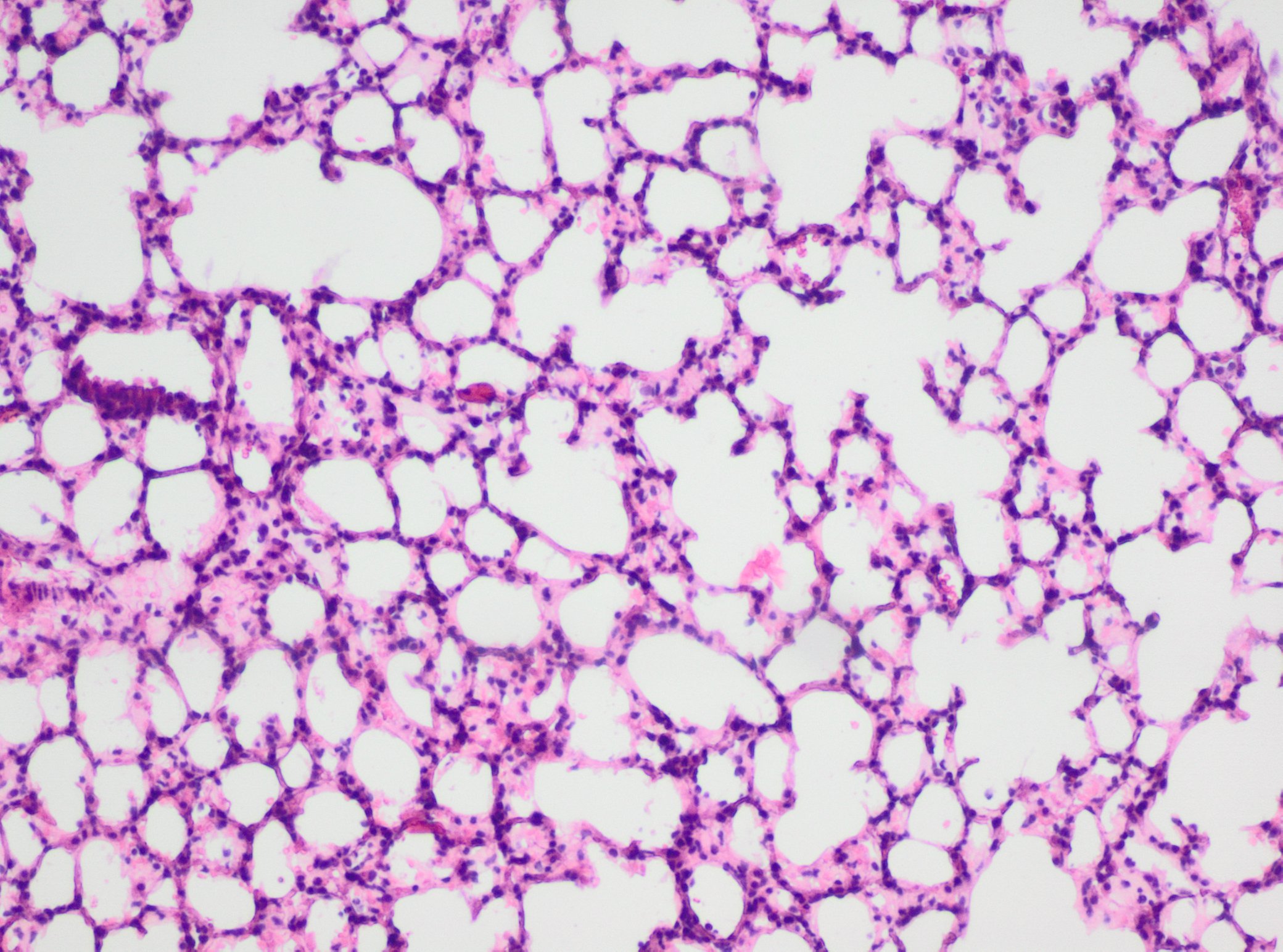**  **Fig. 2b** FiO_2_ 30% | **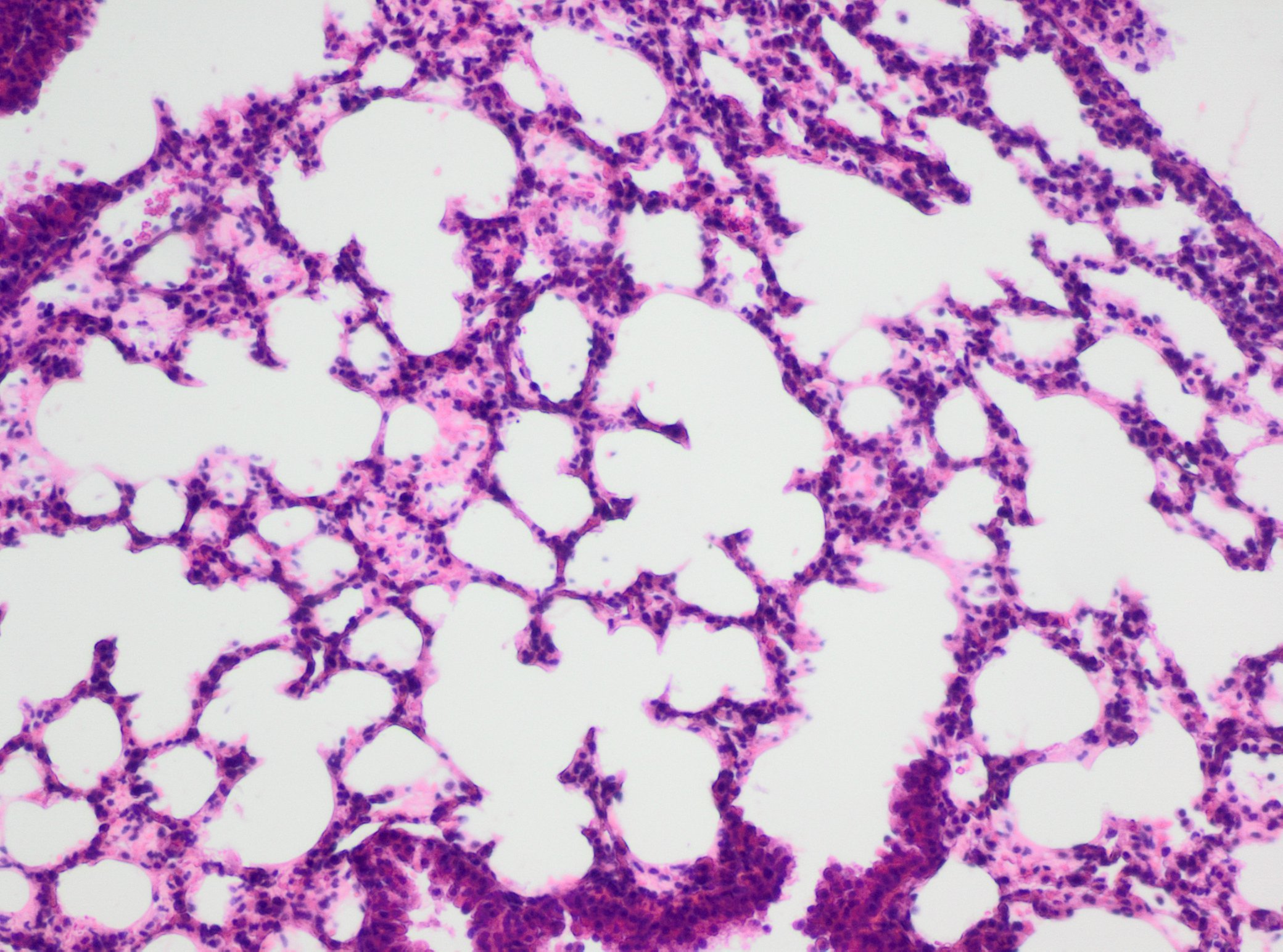**  **Fig. 2c** FiO_2_ 50% | **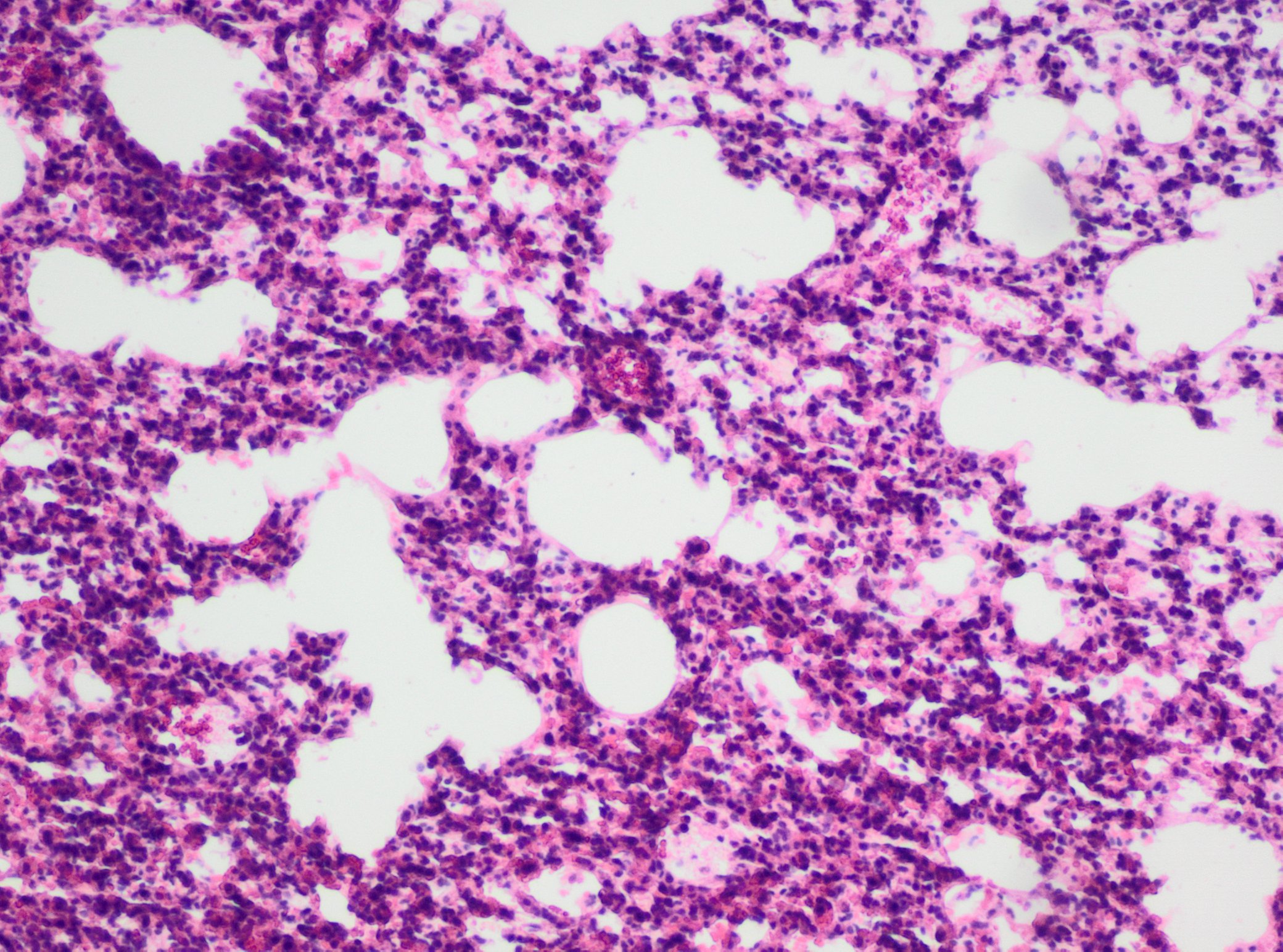**  **Fig. 2d** FiO_2_ 90% |

**Supplemental figure 3.** Inflammatory mediators in BALf after 8 hours of mechanical ventilation

| 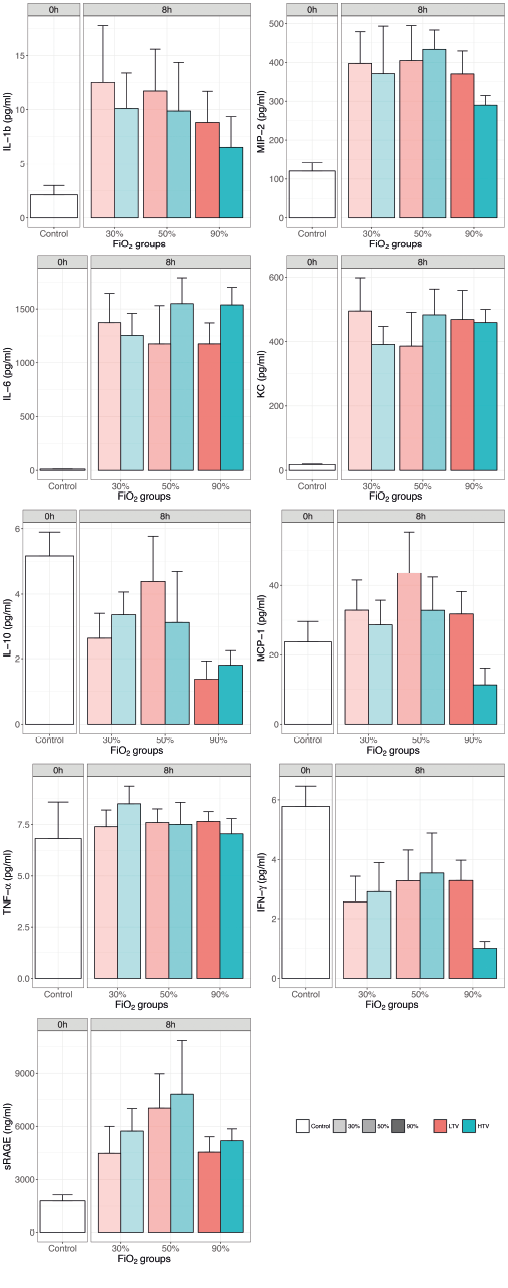 | Cytokine and chemokine levels in BALf obtained after 8 hours of mechanical ventilation. Data are means ±SEM.  Facets within the panels represent mechanical ventilation time. Different colors represent different tidal volume groups and different transparency levels represent different FiO_2_ groups. 0h= no mechanical ventilation time, control group; 12h = 12 hours of mechanical ventilation. LTV= low tidal volumes; HTV= high tidal volumes.  Cuzick’s P for trend in increasing oxygen levels at 12 hours of mechanical ventilation: IL-1β, 0.45; MIP-2, 0.85; IL-6, 0.74; KC, 0.60; IL-10, 0.06; MCP-1, 0.31; TNF-α, 0.44; IFN-γ, 0.71; sRAGE, 0.85 |
| --- | --- |

**Supplemental figure 4.** Inflammatory mediators in BALf after study interval by tidal volume size

| 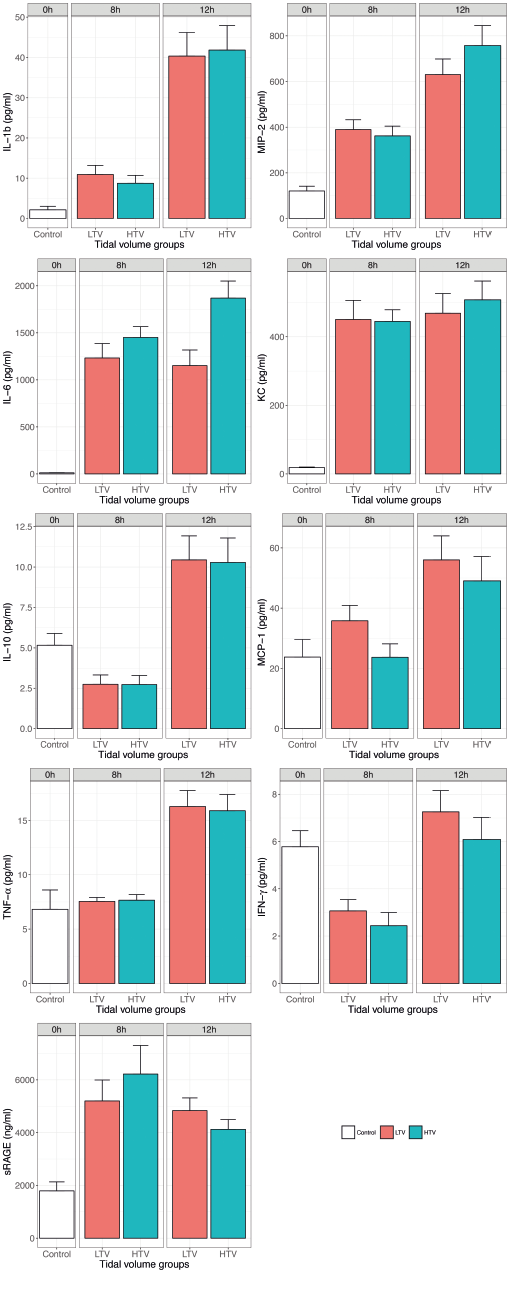 | Cytokine and chemokine levels in BALf obtained after 8 and 12 hours of mechanical ventilation. Data are means ±SEM.  Facets within the panels represent mechanical ventilation time. Different colors represent different tidal volume groups. 0h= no mechanical ventilation time, control group; 8h = 8 hours of mechanical ventilation; 12h = 12 hours of mechanical ventilation. LTV= low tidal volumes; HTV= high tidal volumes. |
| --- | --- |

**Supplemental figure 5.** Inflammatory mediators in serum after 12 hours of mechanical ventilation

| 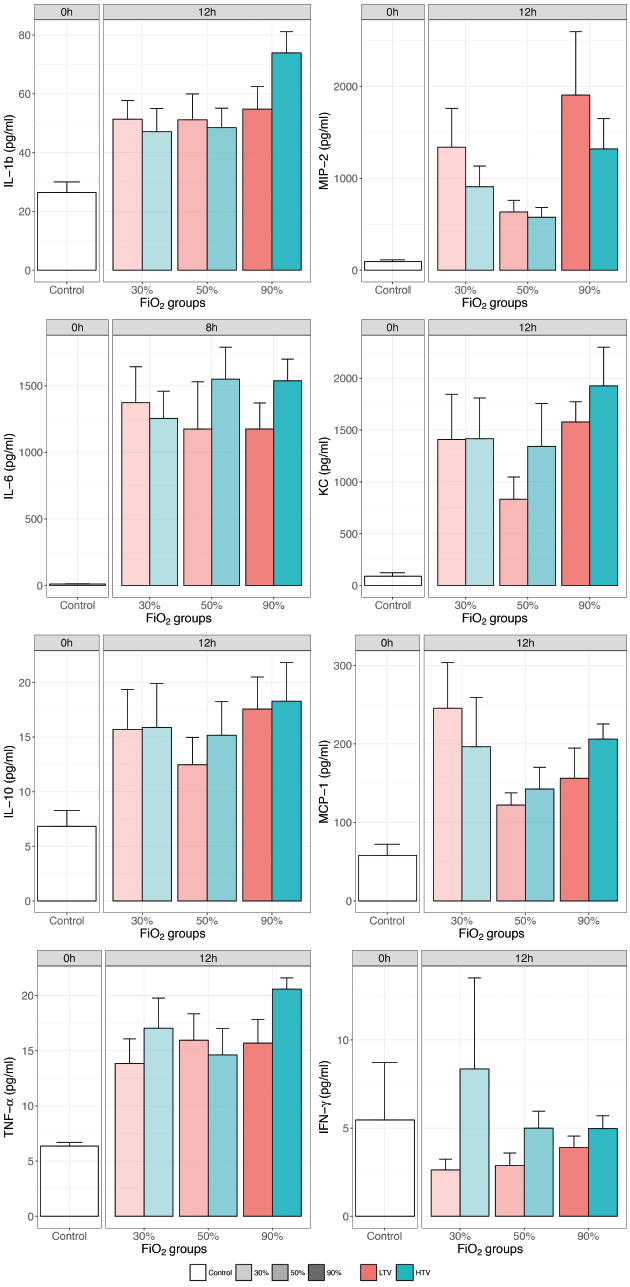 | Cytokine and chemokine levels in serum obtained after 12 hours of mechanical ventilation. Data are means ±SEM.  Facets within the panels represent mechanical ventilation time. Different colors represent different tidal volume groups and different transparency levels represent different FiO_2_ groups. 0h= no mechanical ventilation time, control group; 12h = 12 hours of mechanical ventilation. LTV= low tidal volumes; HTV= high tidal volumes.  Cuzick’s P for trend in increasing oxygen levels at 12 hours of mechanical ventilation: IL-1β, 0.07; MIP-2, 0.47; IL-6, 0.07; KC, 0.15; IL-10, 0.29; MCP-1, 0.87; TNF-α, 0.46; IFN-γ, 0.10 |
| --- | --- |

**Supplemental figure 6.** Relative RNA expression of inflammatory markers in lung homogenate after 12 hours of mice that were mechanically ventilated with high tidal volumes compared to controls
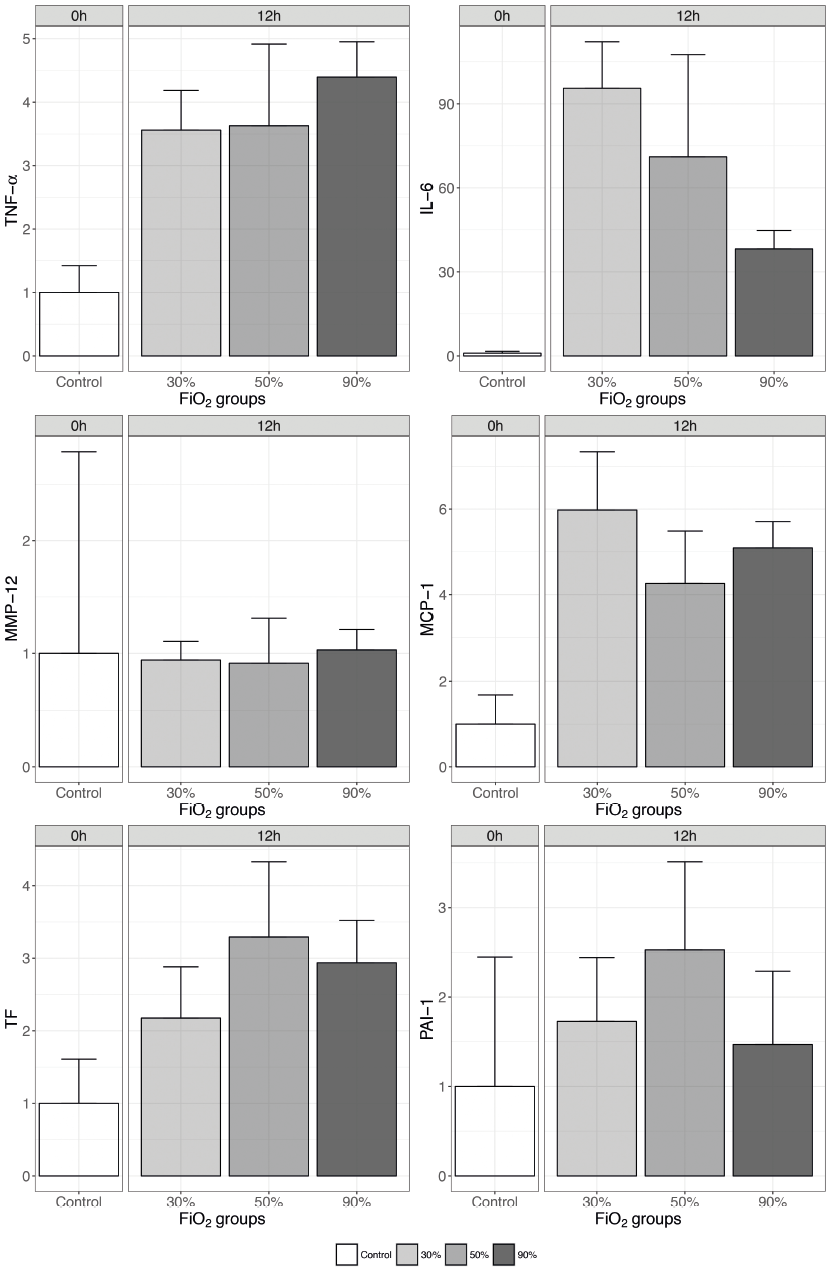


Relative RNA expression of inflammatory markers in lung homogenate obtained after 12 hours of mechanical ventilation. Data are means ±SEM. Facets within the panels represent mechanical ventilation time. Different grayscale transparency levels represent different FiO_2_. 0h= no mechanical ventilation time, control group; 12h = 12 hours of mechanical ventilation.

Cuzick’s P for trend in increasing oxygen levels at 12 hours of mechanical ventilation: TNF-α, 0.33; IL-6, 0.06; matrix metallopeptidase 12 (MMP-12), 0.61; MCP-1, 0.87; tissue factor (TF), 0.60; plasminogen activator inhibitor 1 (PAI-1), 0.72
